# The percutaneous trigger finger release scalpel - the A knife

**DOI:** 10.1186/1753-6561-9-S3-A78

**Published:** 2015-05-19

**Authors:** Sittichoke Anuntaseree

**Affiliations:** 1 Department of Orthopaedic Surgery, Prince of Songkhla University, Songkhla, 90110, Thailand

## 

Open A1 pulley release is a standard surgical procedure for treatment of trigger finger. The disadvantages of the open technique include injury to the soft tissue, developing a painful palmar scar and patients requiring an extended recovery timethesince the procedure is more complex. Another technique used for treatment of trigger finger is percutaneous release. This technique offers the benefits of smaller incision, faster recovery time and an easier procedure compared tothe open technique. The A-Knife is a new specially designed invention for percutaneous trigger finger release. It is made from stainless steel and has these special features for the follow purposes.

1. The scalpel’s size, angle, curve, and position of the blade are designed to cut only the A1 pulley through a 2 millimeter wound on the skin.

2. The tip of the A-Knife has a round end which is used to guide through the small skin incision and locating the A1 pulley with minimal injuries to the tendon and surrounding tissues, allowing the surgeon to precisely locate the problematic landmark.

3. The curve of the scalpel is appropriate to make effective insertion the tip of the scalpel between the A1 pulley and the flexor tendon. The angle of the scalpel is tailored to cut the A1 pulley in a proper direction. Results of A1 pulley release by the A-Knife show a reduction in injuries to the surrounding soft tissue and lessened risk of the injury to the tendon.

There are 6 steps in the surgical technique of percutaneous trigger finger release using the A-Knife. (Figure 1)

**Figure 1:**
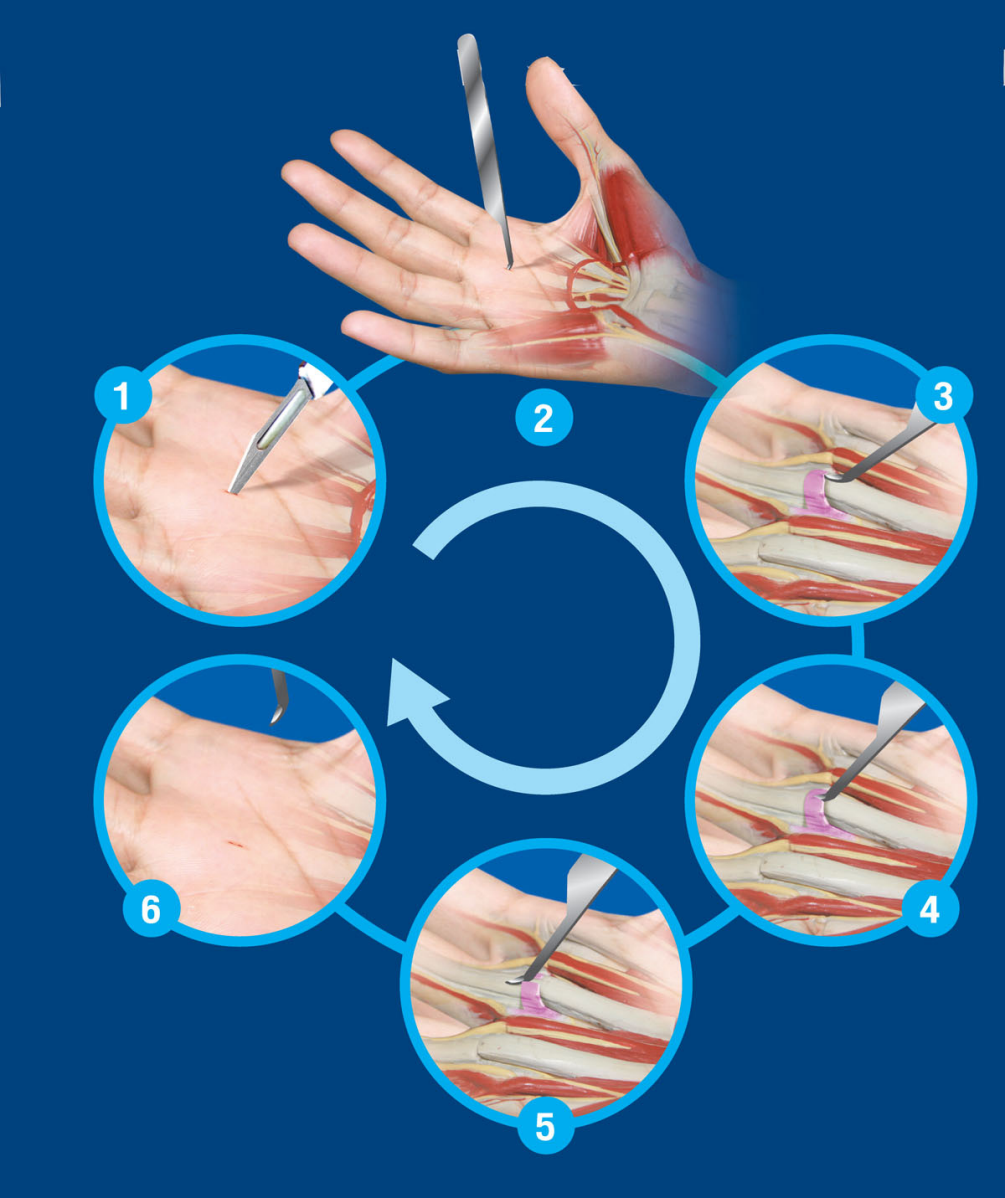
The surgical technique of percutaneous release trigger finger using the A-Knife.

1. The local anesthesia is injected and makes a 2-millimeter incision on the skin.

2. The A-Knife is inserted through the incision.

3. The edge of A1 pulley is identifyby the blunt tip of the A-Knife.

4. A surgeon inserts and passes the tip of the A-Knife under the tendon sheath.

5. After the tip of the A-Knife is in a position between the A1 pulley and tendon, the A-Knife is passed distally parallel to the tendon to cut the A1 pulley.

6. The A-Knife is removed from the incision. The finger motion without locking or snapping should be reach at this end step.

## Summary

A-Knife is a scalpel specially designed for the minimally invasive surgical treatment of a trigger finger. The advantages of the procedure are short operative time, safety and ease as an office procedure. The patient will have a rapid recovery period and less post-operative pain.

